# Severe and Late Acute Liver Injury Induced by Capecitabine

**DOI:** 10.7759/cureus.12477

**Published:** 2021-01-04

**Authors:** Mhd Baraa Habib, Ibrahem Hanafi, Maya Al Zoubi, Zeina Bdeir, Mohamed A Yassin

**Affiliations:** 1 Internal Medicine, Hamad Medical Corporation, Doha, QAT; 2 Internal Medicine, Faculty of Medicine, Damascus University, Damascus, SYR; 3 Hematology, Hamad Medical Corporation, Doha, QAT

**Keywords:** capecitabine, severe hepatotoxicity, late acute liver injury, colon cancer, reversible adverse effect

## Abstract

Capecitabine (CAP) is an antineoplastic agent that is known to cause mild hepatotoxicity. However, severe and late acute liver injury was not reported previously as an adverse reaction of CAP. This report discusses the case of a 63-year-old man with colon cancer who was receiving the fifth cycle of CAP as a monotherapy and presented with fatigue and jaundice during the fifth cycle of CAP. Laboratory tests showed markedly elevated transaminases (aspartate transaminase: 2,448 U/L; alanine transaminase: 1,984 U/L). Eventually, discontinuation of CAP was enough to reverse the delayed CAP-induced acute hepatic injury in clinical and laboratory terms.

## Introduction

Drug-induced liver injuries can be categorized into five patterns: cholestatic hepatitis, acute hepatitis, chronic hepatitis, chronic cholestasis, and acute cholestasis [1]. The American DILI Network reported that antibiotics, herbal agents, cardiovascular agents, anti-neoplastic agents, analgesics, and many other classes are implicated in drug-induced liver injury [2]. Colorectal cancer is one of the most common and fatal malignancies worldwide [3]. Capecitabine (CAP), an oral prodrug of 5-fluorouracil, is a pyrimidine analogue that has been used since 1998 to treat advanced colon cancer [4]. The recommended regimen of CAP is 850-1,250 mg/m^2^ orally twice daily for 14 days. The cycle needs to be repeated every three weeks for total eight cycles [5]. The most common adverse reactions are diarrhea, nausea, vomiting, abdominal pain, fatigue/weakness, and hyperbilirubinemia [6]. Drug-induced liver injury is a known side effect of CAP therapy, which usually manifests with a high bilirubin level. The suggested mechanism is thought to be direct hepatotoxicity. CAP is mainly metabolized in the liver through the microsomal enzyme system, and production of toxic substances may induce liver injury [4]. Serum aminotransaminase rarely may increase in some patients receiving CAP; however, it is unusual to result in high levels more than five times of upper limit of the reference range [4].

In this case report, we present the case of an elderly man who was treated with CAP for colon cancer and presented to our center in the fifth cycle of CAP because of acute liver injury with elevated transaminase in thousands.

## Case presentation

A 63-year-old male patient was diagnosed with colorectal cancer accidentally after urgent surgery for bowel obstruction. He was referred to the oncology center and received four cycles of adjuvant chemotherapy with a conventional dose of CAP at 1,250 mg/m^2^. He was on the 10th day of the fifth cycle when he presented to our hospital complaining of fatigue and yellowish color of his eyes for one week. The patient denied any fever or abdominal pain but mentioned clay-colored stool and dark-colored urine without any changes in defecation or urination habits. Except for the mentioned cancer, his medical history was unremarkable. He also denied any recent intake of alcohol or regular medications or herbals.

On admission, he was afebrile, with a blood pressure of 135/84 mmHg, heart rate of 76 beats per minute, respiratory rate of 17 breaths per minute, and BMI of 26. Physical examination was positive for jaundice noted in sclera and skin. Abdominal examination was unremarkable with no organomegaly. Laboratory findings were significant for markedly elevated serum transaminases and bilirubin (Table 1). Viral hepatitis was ruled out by negative serology for hepatitis A, B, C, and E, Epstein-Barr virus, cytomegalovirus, and herpes simplex virus. Autoimmune panel including ANA, ANCA, and AMA was negative. Ultrasound of the abdomen was unremarkable. CT of the abdomen revealed no hepatic metastases. One month before the presentation, the patient’s baseline liver enzymes and bilirubin were normal (Table 1). The multidisciplinary team decided to discontinue CAP as it was the likely implicated reason behind this severe hepatotoxicity. Two weeks later, the patient’s transaminase level went down with clinical improvement as well. Therefore, the diagnosis of CAP-induced acute liver injury was established. Laboratory tests after six-month follow-up revealed normal serum transaminase and bilirubin levels. At that point, the patient was not taking any chemotherapy and he refused to resume chemotherapy afterward.

**Table 1:** Blood tests ALT, alanine transaminase; AST, aspartate transaminase; INR, international normalized ratio

Detail	One month before the presentation	On admission	Two weeks after hospitalization	Normal range
ALT	16	1,984	110	1-43 U/L
AST	21	2,448	68	1-43 U/L
Total bilirubin	0.97	23.03	3.96	0.5-1.2 mg/dL
Direct bilirubin	NA	20.8	2.10	0.00-0.30 mg/dL
Alkaline phosphatase		523		90-290 U/L
INR		1.25		0.9-1.2
Urea		34		10-40 mg/dL
Creatinine		1.09		0.5-1.4 mg/dL
Sodium		141		136-145 mmol/L
Potassium		4.1		3.5-5.1 mmol/L

## Discussion

Severe hepatotoxicity (high alanine transaminase [ALT] and aspartate transaminase [AST] > 1,000 U/L) is usually related to a few etiologies including viral hepatitis, ischemic liver injury, and toxin- or drug-induced liver injury [7]. Drug-induced liver injury is a common cause of acute liver injury in general population. A detailed history taking should focus on the possible hepatotoxic medications along with other potential etiologies [8]. Although CAP is mainly metabolized by the liver, hepatopathy due to CAP is not a frequent manifestation due to its rapid metabolism and short half-life [9]. Mild hyperbilirubinemia is a well-known side effect of CAP. However, it is often reversible and isolated without other abnormal liver tests [4]. In addition, one article reported a mild serum enzyme elevation that was accompanied by steatosis and inflammation in a patient treated with CAP. This pathology resolved after holding the chemotherapy [10]. Hence, even though several hepatic adverse effects were reported, the severely elevated transaminase was not noticed.

In our case, the patient developed a late elevation of AST and ALT (while he was receiving the fifth course of CAP), which was not noticed in the previous cycles. Furthermore, it was a very severe increment in transaminase (in thousands), which was not reported previously in association with CAP as the patient was vitally stable and the viral hepatitis and autoimmune panels were negative; ischemic and viral etiologies of severe transaminitis were excluded. Therefore, drug-induced hepatotoxicity emerged as the principal diagnosis. A liver biopsy for histological confirmation was not performed in our patient due to the complete recovery of liver enzyme abnormalities after discontinuation of CAP. These unique associated findings were not reported previously as an adverse reaction of CAP.

## Conclusions

CAP-induced acute liver injury with very high transaminase can be a serious and late side effect, and it should be considered anytime during the course of treatment. Monitoring liver enzymes for possible hepatotoxicity of CAP and the immediate cessation of treatment might be advisable to mitigate the toxic effects and possible complications.
